# Spatiotemporally random and diverse grid cell spike patterns contribute to the transformation of grid cell to place cell in a neural network model

**DOI:** 10.1371/journal.pone.0225100

**Published:** 2019-11-14

**Authors:** Sahn Woo Park, Hyun Jae Jang, Mincheol Kim, Jeehyun Kwag

**Affiliations:** Neural Computational Laboratory, Department of Brain and Cognitive Engineering, Korea University, Seoul, Korea; Georgia State University, UNITED STATES

## Abstract

The medial entorhinal cortex and the hippocampus are brain regions specialized in spatial information processing. While an animal navigates around an environment, grid cells in the medial entorhinal cortex spike at multiple discrete locations, forming hexagonal grid patterns, and each grid cell is spatiotemporally dynamic with a different grid size, spacing, and orientation. In contrast, place cells in the hippocampus spike when an animal is at one or more specific locations, called a “place field”. While an animal traverses through a place field, the place cell’s spike phases relative to the hippocampal theta-frequency oscillation advance in phase, known as the “spike phase precession” phenomenon and each spike encodes the specific location within the place field. Interestingly, the medial entorhinal cortical grid cells and the hippocampal place cells are only one excitatory synapse apart. However, how the spatiotemporally dynamic multi-peaked grid cell activities are transformed into hippocampal place cell activities with spike phase precession phenomenon is yet unknown. To address this question, we construct an anatomically and physiologically realistic neural network model comprised of 10,000 grid cell models, each with a spatiotemporally dynamic grid patterns and a place cell model connected by excitatory synapses. Using this neural network model, we show that grid cells’ spike activities with spatiotemporally random and diverse grid orientation, spacing, and phases as inputs to place cell are able to generate a place field with spike phase precession. These results indicate that spatiotemporally random and diverse grid cell spike activities are essential for the formation of place cell activity observed *in vivo*.

## Introduction

**Fig 1:**
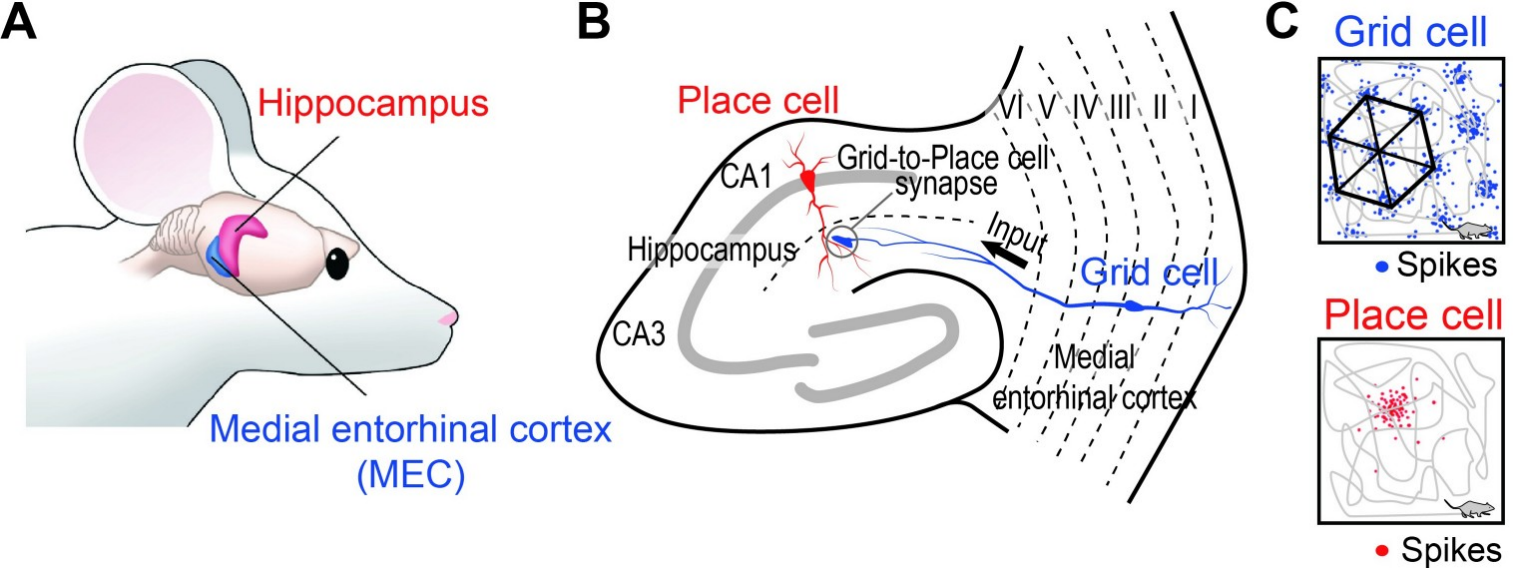
Place cell in the hippocampus and grid cell in the medial entorhinal cortex of the rodent brain. (A) Anatomical location of the hippocampus and the medial entorhinal cortex (MEC) in the rodent brain. (B) Anatomical neural circuit of the hippocampus and the MEC. Grid cell (blue) in layer III of the MEC provides direct synaptic input to place cell (red) in the hippocampus. (C) Examples of spiking patterns of a grid cell (top, blue) and a place cell (bottom, red) while a rodent navigates (trajectory of a rodent: gray line) around a 1 m × 1 m square environment (black square). A grid cell generates spikes (blue dots) at multiple locations, forming haxagonal grid patterns (black hexagon) called “grid fields” (top) while a place cell generates spikes (red dots) selectively at one or more specific locations (bottom) called a place field.

The ability to locate one’s current position and to navigate around in the external environment is critical for survival. The medial entorhinal cortex (MEC) and the hippocampus of the mammalian brain are known to be the centers for such spatial information processing (Fig 1A). There, two different types of spatially-selective neurons have been identified: grid cells in the MEC [1–3] and place cells in the hippocampus [4–6] (Fig 1B). MEC grid cells show increased spike firing rate at multiple discrete locations called “grid fields” (Fig 1C, top) and these multi-peaked grid fields are arranged to form hexagonal grid-like firing patterns that are regularly spaced over the entire environment [1]. By maintaining a positional relationship with the environment that is independent of the contextual information [7], grid fields effectively act as grid coordinates of the environment. These grid field patterns are spatiotemporally diverse and dynamic, where grid field size and grid spacing (distance between individual grid fields) increase as the grid cell’s anatomical location moves from the dorsal to ventral part of the MEC [8]. In addition, grid orientation (the rotation of grid axes) and the grid phase (x-y axis of the firing vertices) [1, 9, 10] can be changed. Such spatiotemporally diverse and dynamic grid cell characteristics allow for multiple grid cells to code the entire environment [1, 9].

In contrast, place cells in the CA1 area of the hippocampus typically spike at one or more discrete locations in the environment called “place fields” [5, 6, 11, 12] (Fig 1C, bottom). Interestingly, when the rat traverses through the place field, the spike phases of a place cell relative to the ongoing theta-frequency oscillation progressively advance 360° over each theta cycle [6, 13]. Such a phenomenon, which is called a “spike phase precession”, demonstrates that spike firing rate codes the place field within the environment, while each spike phase codes for the specific location of an animal within the place field [6].

Although these two types of neurons have distinct functional roles in spatial information processing, how such spiking characteristics arise in the neural network of the brain is still unclear. Anatomically, grid cells located in layer III of the MEC directly project their axons to place cells in the CA1 area of the hippocampus [14] (Fig 1B). Thus, being only a single synapse apart, grid cells have naturally been assumed to be a precursor of place cells [15, 16]. Indeed, bilateral MEC lesions disrupted the spike phase precession in CA1 place cells [17]. Moreover, pharmacological [18], surgical [19], optogenetic [20], and chemogenetic [21] blockade of synaptic transmission between the MEC and the CA1 area of the hippocampus impaired hippocampal place cell activity. These studies strongly suggest that inputs from the MEC are critical for the generation of a place cell. However, it is still a mystery how multi-peaked hexagonal grid spike patterns of grid cells in the MEC could be transformed into spike activities in specific place fields of hippocampal place cells with spike phase precession, being only a single synapse apart [14, 22].

Simultaneous recordings of anatomically connected MEC grid cells and hippocampal place cells will give us clues to whether MEC grid cells are indeed precursors of hippocampal place cells. However, this experimental technique is currently unavailable. Hence, many theoretical and computational modeling studies have been attempted to overcome the experimental limitation [15, 16, 23–28]. In these models, MEC grid cells with different sizes, spacings, and orientations [16, 23–26], as well as synaptic learning rules [23, 24, 27] have been shown to influence the grid-to-place cell transformation. Moreover, non-spatial inputs [24, 28, 29] and inhibition [30] have also been shown to play roles in grid-to-place cell transformation. Although successful in demonstrating grid-to-place cell transformation, none of these models replicate the other spatial feature of the place cell: spike phase precession [6].

*In vivo* patch-clamp recordings of hippocampal place cells have revealed that place cells receive depolarizing excitatory ramp-like input (ERI) while the rat is in the place field [31], and such ERIs have been shown to cause spike phase precession in hippocampal neurons in *in vitro* experiments [32–34] and in an *in silico* modeling study [33]. This shows that transformation of the MEC grid cell inputs to ERI would dictate whether spike phase precession would occur. Thus, accurate modeling of the physiological and anatomical characteristics of grid cells, place cells, and the synapse between them is required. However, previous computational models investigating grid-to-place cell transformation modeled place cells as reduced integrate-and-fire neurons [30], simplified spiking units [25, 26] or even non-spiking units [16, 35], where the role of place cell models was to simply sum the firing rates of grid cells [16, 25], thus, no ERI nor spike phase precession was observed in these studies. Furthermore, place cells in the CA1 area of the hippocampus receive around 100–1,000 synaptic inputs directly from neurons in the MEC [16, 22, 36]. The inputs from the MEC are spatiotemporally diverse and produce dynamic grid patterns that vary in size, spacing, and orientation, and the MEC axons make excitatory synapses located 300–400 μm from the soma of the place cell in the CA1 area of the hippocampus [14, 22, 37]. These anatomical and physiological details should also be taken into close consideration in order to closely simulate the *in vivo* characteristics of grid-to-place cell transformation.

In this study, we developed a computational neural network model consisting of an *in vivo*-like grid cell model and a multi-compartment Hodgkin-Huxley place cell model connected by anatomically and physiologically realistic excitatory synapses that closely reflects the *in vivo* and *in vitro* recorded grid cell and place cell characteristics. Using this neural network model, we investigated the conditions under which grid cell spike outputs would transform into a place field with ERI at the place cell that can cause spike phase precession [33]. We show that grid cells with spatiotemporally random and diverse grid patterns as inputs to place cells could generate robust grid-to-place cell transformation with spike phase precession, suggesting that random and diverse neural activities could explain spatial information processing in the brain.

## Material and methods

To investigate how spatiotemporally diverse and dynamic hexagonal grid-like spike patterns of grid cells in the MEC could be transformed into spike activities of hippocampal place cells at one or more specific locations with spike phase precession, we built a computational neural network model consisting of MEC grid cells and a hippocampal place cell connected by excitatory synapses.

In order to capture the navigational characteristics of rodents into the computational neural network model of grid cell and place cell, the positional data of a freely moving rodent within a 1 m × 1 m square box obtained from the publicly available dataset (http://www.ntnu.edu/kavli/research/grid-cell-data) was used.

### *In vivo*-like grid cell model

We developed an *in vivo*-like grid cell model by modifying the conventional oscillatory interference (OI) grid cell model [38] to generate spikes in Gaussian manner, as observed *in vivo* [1, 3, 16]. The conventional OI grid cell model realistically replicates the spatially periodic firing patterns of grid cells as a consequence of interference between background theta-frequency oscillation and six velocity-controlled oscillation (VCO) during exploration [9, 38–40]. The frequency of each VCO is determined by the speed of the animal headed in a specific preferred direction (*ϕ*_*pref*_) and each of the six VCOs has preferred directions that are separated by 60° (0, 60, 120, 180, 240, and 300°).

$$f_{VCO_{i}}(t)=f_{theta}+\beta s(t)\left(\cos\left(\emptyset_{pref_{i}}-\emptyset (t)\right)\right)$$ (1)

Here, *f*_*theta*_ is set to 10 Hz. *s*(*t*) and *ϕ*(*t*) are the speed and direction derived from the velocity of the rat, respectively, and *β* is a positive constant that controls the field size and spacing of the grid field. The interference of six VCOs and background thetafrequency oscillation is calculated by *g*(*t*), as in the following equation:

$$\begin{array}{l} g\left(t\right)=\sum_{i=1}^{n_{VCO}}\left(\cos\left(2\pi f_{theta}t\right)+\cos\left(2\pi \left(f_{theta}+f_{VCO_{i}}\right)t\right)\right)\\ =n_{VCO}\cos\left(2\pi f_{theta}t\right)+\sum_{i=1}^{n_{VCO}}\left(\cos\left(2\pi \left(f_{theta}+f_{VCO_{i}}\right)t\right)\right) \end{array}$$ (2)

**Fig 2:**
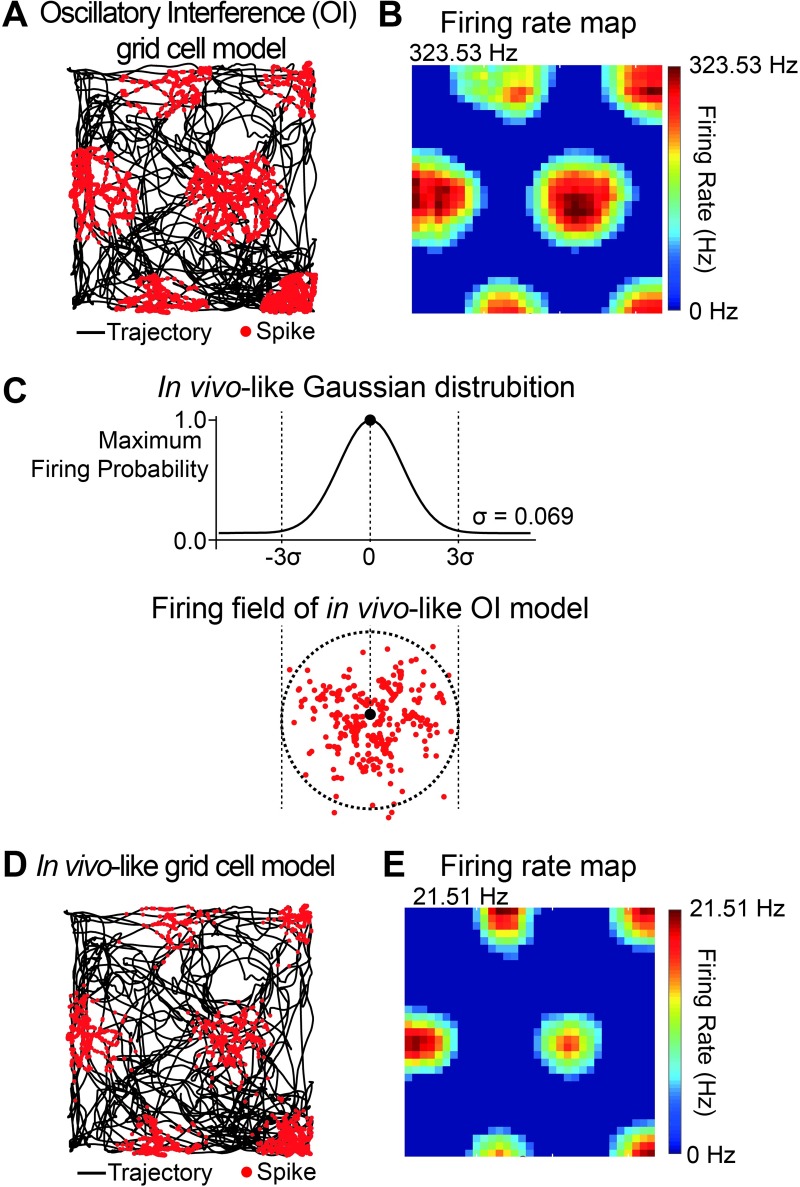
*In vivo*-like oscillatory interference (OI) grid cell model. (A) Trajectory of a rodent navigating within a 1 m × 1 m square environment obtained from *in vivo* recording (black line) and spikes (red dots) simulated with conventional oscillatory interference (OI) grid cell model is superimposed to the trajectory. (B) Firing rate of grid cell in the square environment, called the “firing rate map” (red: peak firing rate (323.53 Hz); blue: no spike (0 Hz)). (C) Gaussian distribution (σ = 0.069, center of grid field to border = 3σ) used to model *in vivo*-like grid field spike pattern (red dots). (D) Spikes (red dots) from *in vivo*-like OI grid cell model plotted over trajectory (black line). (E) Firing rate map of *in vivo*-like OI grid cell model (red: peak firing rate (21.51 Hz); blue: no spike (0 Hz)).

When *g*(*t*) reaches the threshold (*g*_*thres*_ = 1 × the number of VCOs *=* 6), the OI grid cell model is assumed to have generated a spike, resulting in a spatially periodic hexagonal grid cell firing pattern, as seen in Fig 2A.

**Fig 3:**
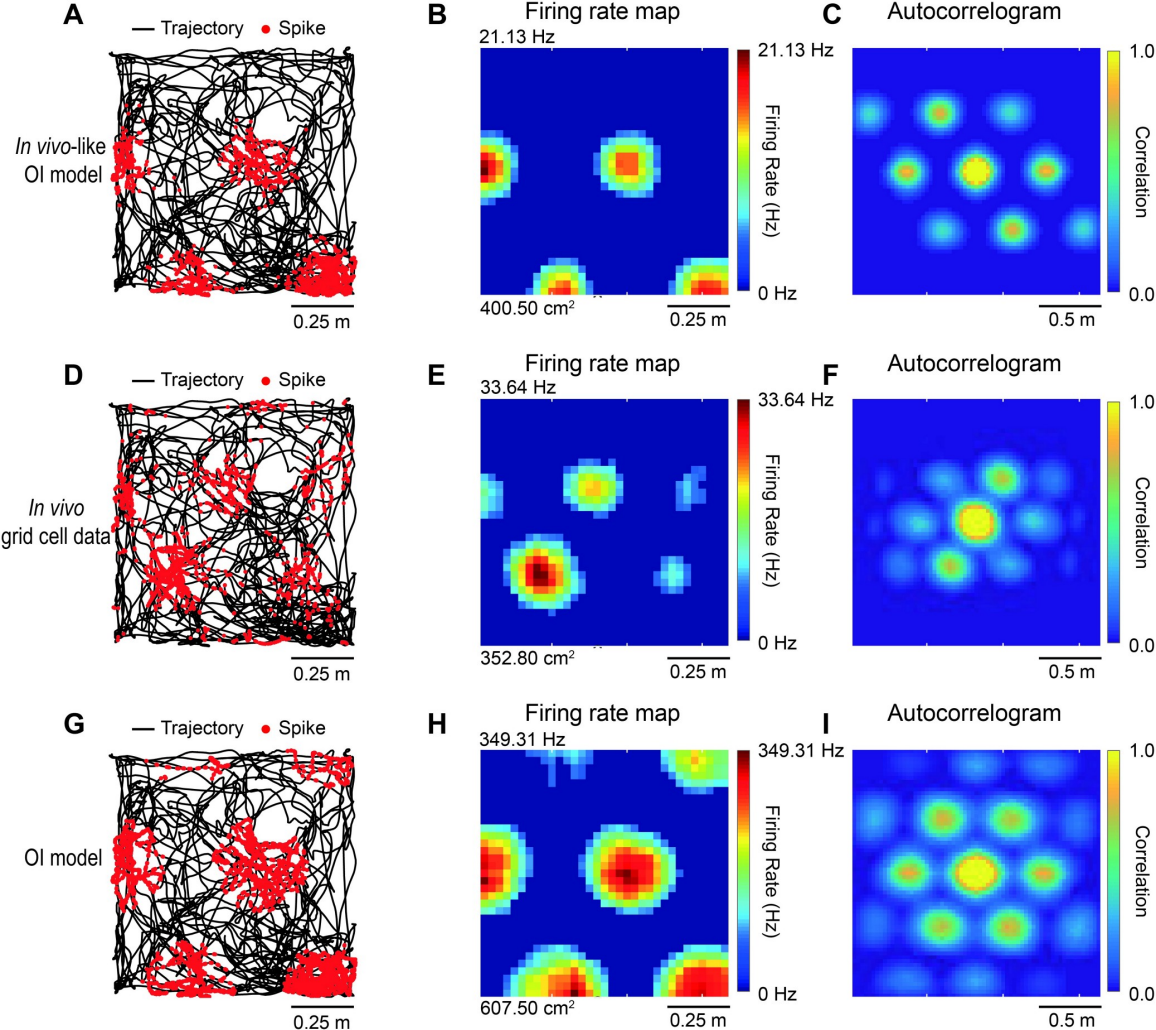
Grid fields of *in vivo*-like OI model, *in vivo* grid cell data, and OI model. (A) Spike (red dots) over trajectory (black line) and (B) firing rate map simulated with *in vivo*-like OI model. (C) The spatial autocorrelogram of firing rate map plotted as color plot (correlation coefficient of 1.0: yellow, correlation coefficient of 0.0: blue). (D-F) Same figures as (A-C), but with *in vivo* grid cell data. (G-I) Same figures as (A-C), but with grid cell spikes simulated with conventional OI grid cell model.

To make the firing rate of the OI grid cell model more *in vivo*-like as observed in *in vivo* experimental studies [1, 3] and a computational modeling study [16] because the firing rate of the OI grid cell model was unphysiologically higher than that of the *in vivo* grid cell data (Figs 2 and 3), we applied a Gaussian probability curve adopted from a Gaussian-approximated place field model [4] to each grid field in the conventional OI model (Fig 2C).

$$p_{Gauss}(t)=\frac{1}{\sigma \sqrt{2\pi }}\exp\left(-\frac{{\left(d(t)-\mu \right)}^{2}}{2\sigma^{2}}\right)$$ (3)

**Fig 4:**
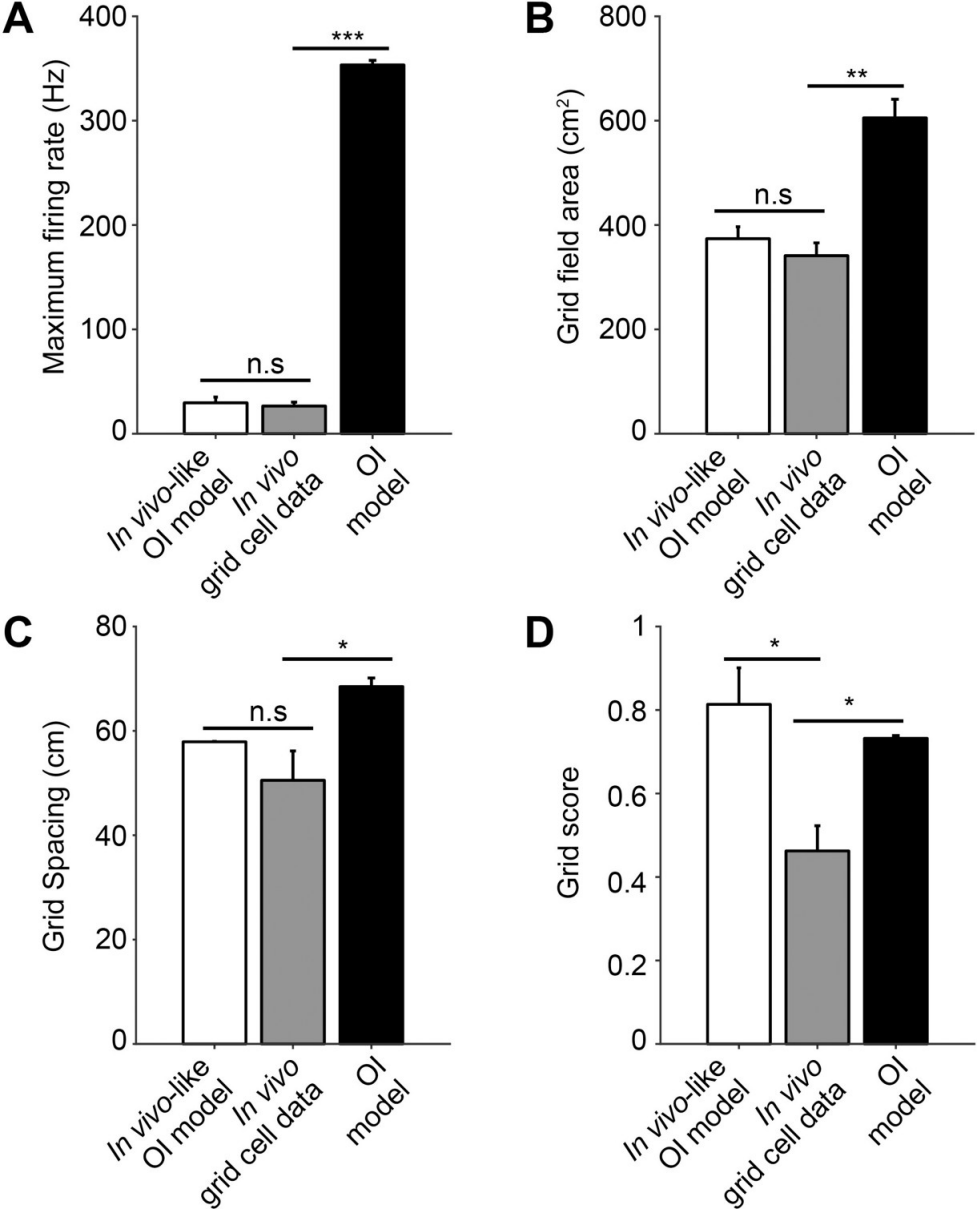
Comparison of *in vivo*-like OI model, *in vivo* grid cell data, and OI model. (A) Maximum firing rate, (B) grid field area, (C) grid spacing between grid fields and (D) grid score of *in vivo*-like OI model (empty), *in vivo* grid cell data (gray), and the OI grid cell model (black). (*: *p* < 0.05, ** < 0.01, *** < 0.001, n.s > 0.05).

*p*_*Gauss*_ is the spike probability at current position *t*; *μ* is the mean value in the Gaussian probability, *d* is the distance between the maximum firing rate location and the nearest center of the firing fields, and σ is the standard deviation set to one-third of the firing field radius to make the firing field size and firing rate similar to the *in vivo* grid cell data (Figs 3 and 4).

**Fig 5:**
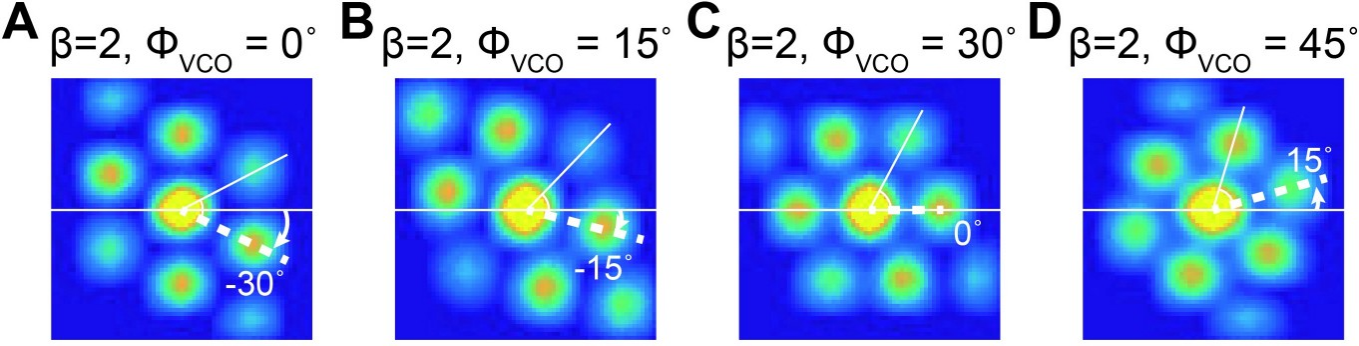
Modulation of grid field orientation of *in vivo*-like OI model by VCO (ϕ_*VCO*_) preferred direction. (A-D) The spatial autocorrelogram of the *in vivo*-like OI model when *ϕ*_*VCO*_ values are (A) 0°, (B) 15°, (C) 30°, and (D) 45° with *β* fixed at 2 Hz/(m/s).

**Fig 6:**
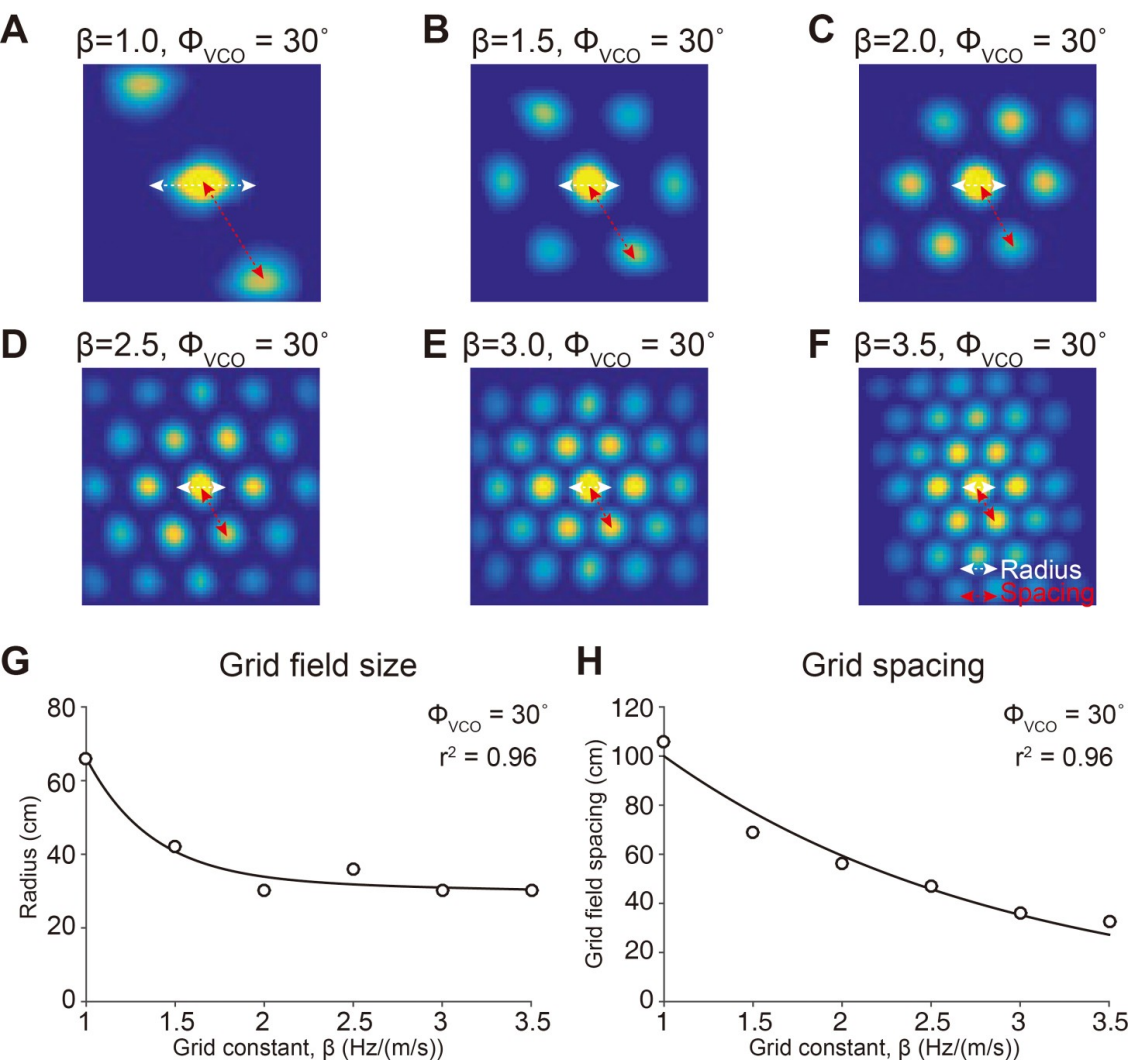
β controls the size and spacing of grid patterns of the *in vivo*-like OI model. (A-F) The spatial autocorrelogram of the *in vivo*-like OI model when *β* values are (A) 1.0, (B) 1.5, (C) 2.0, (D) 2.5, (E) 3.0, and (F) 3.5 Hz/(m/s) with *ϕ*_*VCO*_ fixed at 30°. (G) Grid field size (open circle) plotted as a function of *β*, fitted with an exponential curve (black line, r^2^ = 0.96). (H) Grid field spacing (open circles) plotted as a function of *β*, fitted with an exponential curve (black line, r^2^ = 0.96).

**Fig 7:**
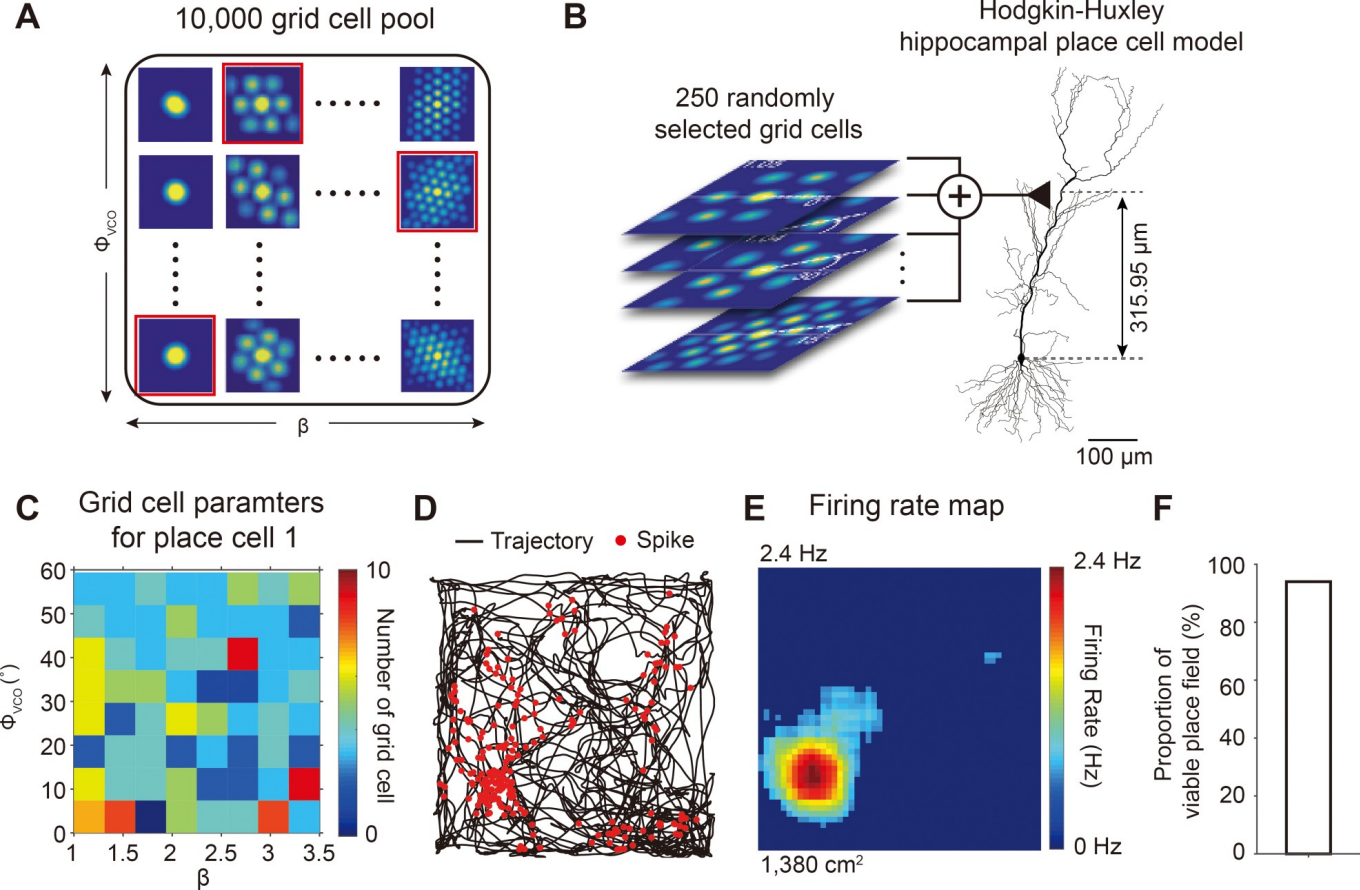
Summation of 250 spatiotemporally random grid cell inputs to single distal dendritic synapse of place cell could generate grid-to-place field transformation. (A) Pool of 10,000 grid cells generated with *in vivo*-like OI grid cell model through varying orientation (*ϕ*_*VCO*_) and spacing (*β*). (B) 250 grid cells, each with different spatiotemporal grid field patterns, were randomly selected from the pool and were used to generate excitatory inputs to Hodgkin-Huxley hippocampal place cell model through a synapse located 315.95 μm from the soma. (C) Distribution of (*ϕ*_*VCO*_, *β*) of 250 randomly selected grid cells (color bar: number of grid cells). (D) Spikes of Hodgkin-Huxley place cell model (red dots) plotted over trajectory (black line). (E) Firing rate map of the Hodgkin-Huxley place cell model. (F) The proportion of viable place fields generated by 100 different sets of grid field patterns, each set containing 250 spatiotemporal random grid fields patterns.

**Fig 8:**
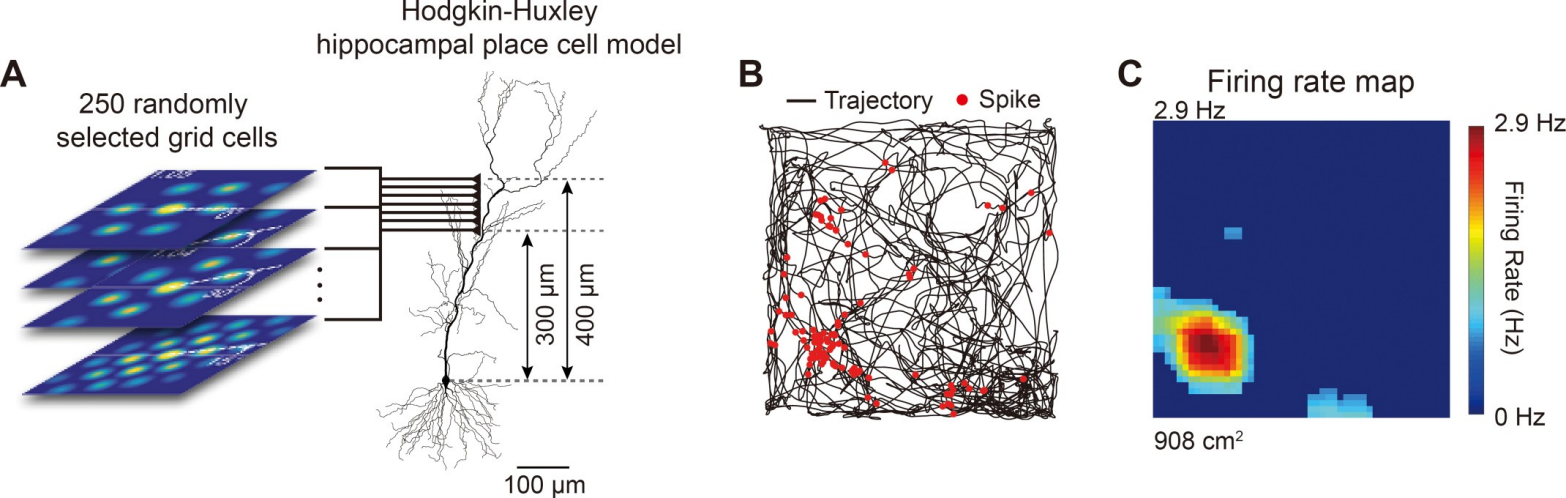
Summation of 250 spatiotemporally random grid cell inputs to spatially distributed synapses in distial dendrites of place cell model could generate grid-to-place field transformation. (A) 250 grid cells, each with different spatiotemporal grid field patterns, were randomly selected from the pool and were used to generate excitatory inputs to Hodgkin-Huxley hippocampal place cell model through 250 excitatory synapses spatially distrubted at 300–400 μm from the soma. (B) Spikes of Hodgkin-Huxley place cell model (red dots) plotted over trajectory (black line). (C) Firing rate map of the Hodgkin-Huxley place cell model.

**Fig 9:**
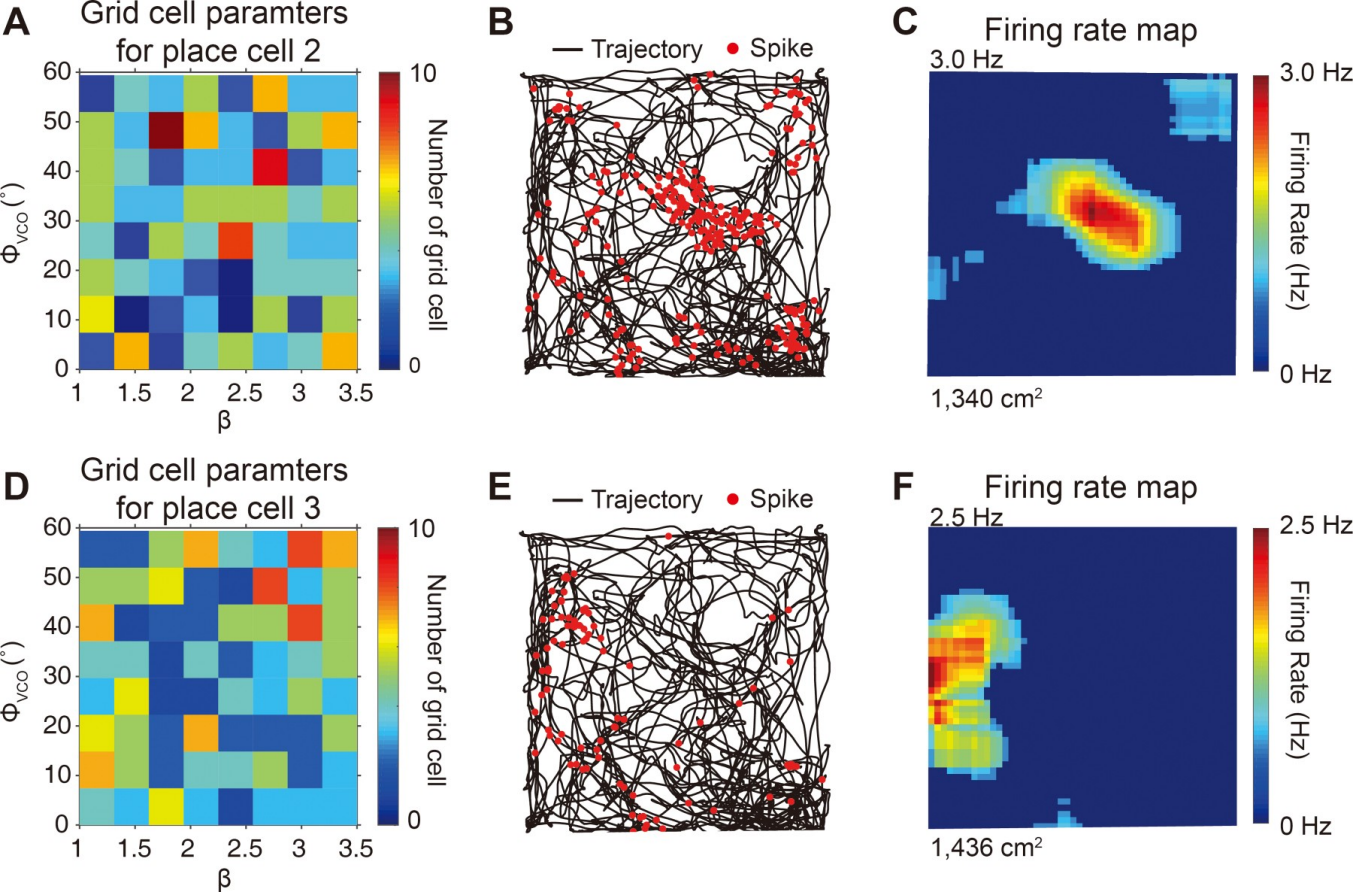
Different place fields generated by different sets of random grid cell inputs. (A) 250 grid cells that were randomly selected from the pool of 10,000 grid cells and each having different distribution of (*ϕ*_*VCO*_,*β*) (Color bar: number of grid cells). (B) 250 grid cells in (A) were used as inputs to the Hodgkin-Huxley place cell model and the resulting place cell spikes (red dots) are plotted over trajectory (black line). (C) Firing rate map of place cell model of (B). (D-F) Same as (A-C) but with place cell receiving different sets of 250 randomly selected grid cells having different distribution of (*ϕ*_*VCO*_,*β*). Note that place fields in (C) and (F) are at different locations.

**Fig 10:**
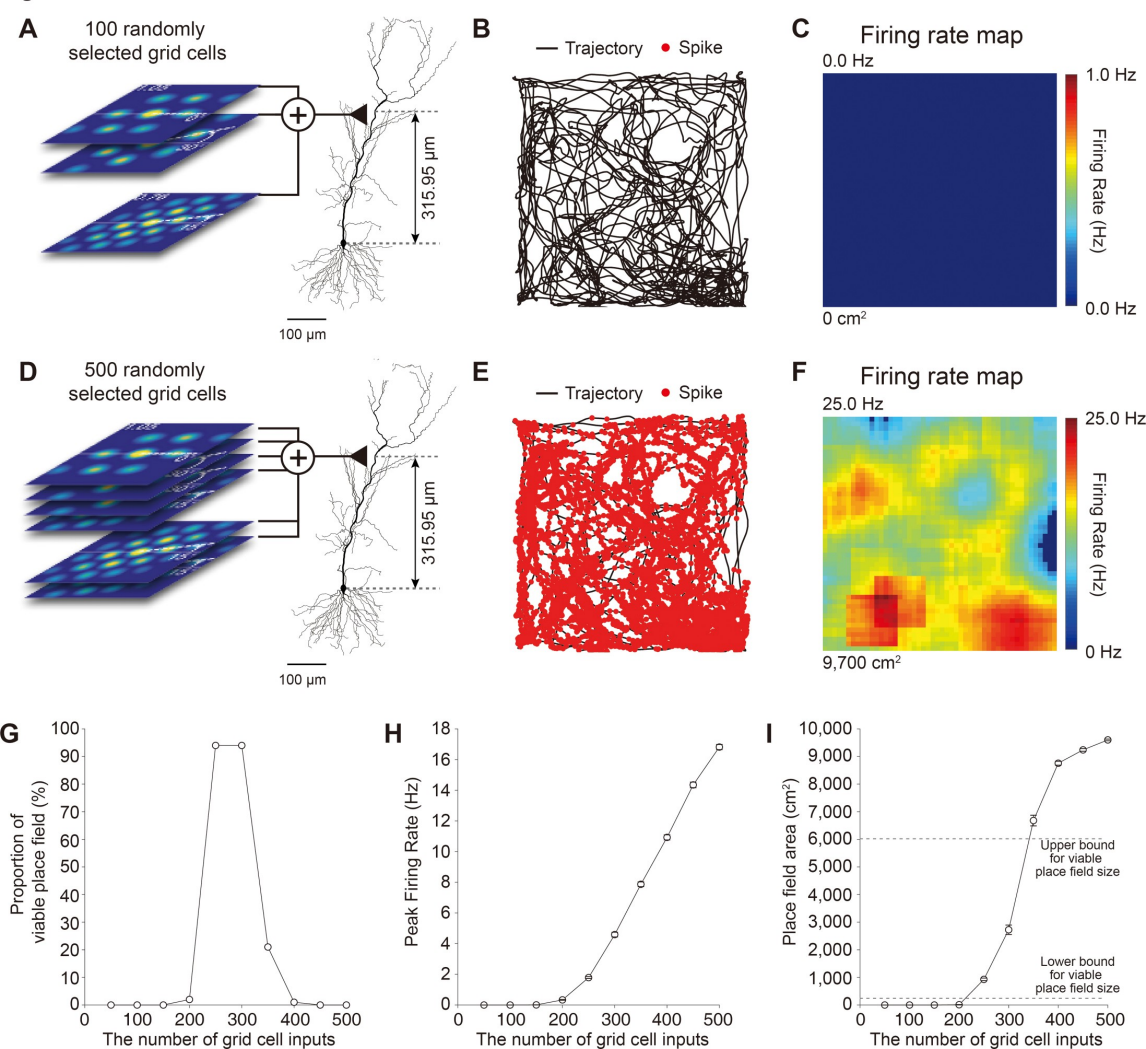
A sufficient number of grid cell inputs is needed for grid-to-place cell transformation. (A) 100 grid cells, each with different spatiotemporal grid field patterns, were randomly selected from the pool of 10,000 grid cells and used as excitatory inputs to the Hodgkin-Huxley hippocampal place cell model. (B) Place cell spikes (red dots) plotted over trajectory (black line). (C) Firing rate map of place cell model of (A). (D-F) Same as (A-C) but place cells receiving inputs from 500 randomly selected grid cells. (G-H) The proportion of viable place fields (G), and peak firing rate as a function of the number of the grid cells (H). (I) Place field area plotted as a function of the number of grid cells. The top gray horizontal dotted line denotes the upper boundary for determining viable place field size (60% of total arena size, 6,000 cm^2^) and the bottom horizontal gray dotted line denotes the lower boundary for determining viable place field size (15 adjacent bins, 240 cm^2^).

In order to capture the spatiotemporally diverse and dynamic grid patterns, we generated a pool of 10,000 grid cells that each fires with different firing patterns by varying *β* and *ϕ*_*pref*_ uniformly within the ranges of 1 ≤ *β* ≤ 3.5 and 0 ≤ *ϕ*_*pref*_ < 60 (Figs 5 and 6) in the *in vivo*-like OI model. From the pool of 10,000 grid cells, 50–500 grid cells were randomly selected as inputs to the place cell model (Figs 7–10). We analyzed the randomness of the grid cell parameter space (*β*, *ϕ*_*pref*_) by calculating the relative ratio of entropy of randomly chosen parameter space (*β*, *ϕ*_*pref*_) to the maximal entropy of parameter space (*β*, *ϕ*_*pref*_) of grid cells (Fig 7). Here, the ratio of entropy indicates the randomness of the chosen parameter set (Eq 4).

$$\begin{array}{l} Randomness=E_{x\in \left\{\beta ,\phi_{pref}\right\}}/Max(E_{x\in \left\{\beta ,\phi_{pref}\right\}})\\ E_{x\in \left\{\beta ,\phi_{pref}\right\}}=-\sum \left(p\left(x\right)\times \log_{2}p\left(x\right)\right) \end{array}$$ (4)

### Hodgkin-Huxley place cell model

The hippocampal place cell was modeled using the Hodgkin-Huxley conductance-based model with 155 compartments that was previously used in other study [33].

$$\begin{array}{c} C_{m}\frac{dV_{m}}{dt}=-({\bar{g}}_{L}(V_{m}-E)+{\bar{g}}_{Na}m^{3}hs(V_{m}-E)+{\bar{g}}_{KDR}n(V_{m}-E)+{\bar{g}}_{KM}n(V_{m}-E)\\ +{\bar{g}}_{KA}nl(V_{m}-E)+{\bar{g}}_{h}l(V_{m}-E)) \end{array}$$ (5)

*C*_*m*_ is the membrane capacitance and *V*_*m*_ is the membrane potential. The model had leak (maximal conductance, ${\bar{g}}_{L}$ = 12.5 *μS*/*cm*^2^), voltage-gated Na^+^ (${\bar{g}}_{Na}$ = 9.4 *mS*/*cm*^2^), delayed-rectifier K^+^ (${\bar{g}}_{KDR}$ = 1.05 *mS*/*cm*^2^), M-type K^+^ (${\bar{g}}_{KM}$ = 45 *μS*/*cm*^2^), A-type K^+^ (${\bar{g}}_{KA}$ = 1.04 *mS*/*cm*^2^) and hyperpolarization-activated (${\bar{g}}_{h}$ = 5 *μS*/*cm*^2^) conductances with a channel kinetics (gating variables: *m*, *h*, *s*, *n*, *l*) and distributions from a previously published model [41].

To simulate the hippocampal theta-frequency oscillation in place cells during the navigation [6, 13, 42, 43], inhibitory conductance (*g*_*inh*_(*t*)) oscillating at a slightly high theta-frequency (10 Hz) that was observed in individual place cells [6, 13, 44, 45] was injected onto the soma of the Hodgkin-Huxley place cell, given that hippocampal theta-frequency oscillation is generated by inhibitory interneurons [46].

$$I_{inh}(t)=\sin\left(2\pi ft\right)\times g_{inh}(t)\;[V_{m}(t)-E_{Rev}]$$ (6)

*I*_*inh*_(*t*) is the current to be injected onto the soma of the place cell model to simulate theta-frequency oscillation, *f* is the frequency set to 10 Hz, *V*_*m*_(*t*) is the membrane potential and *E*_*Rev*_ is the reversal potential of inhibitory oscillating conductance set to -70 mV. Step current was simulated to the Hodgkin-Huxley place cell to sustain a minimum mean firing rate of 2.5 Hz in the place cell [47].

### Excitatory synapse model

The excitatory synaptic inputs between the grid firing patterns from a grid cell and a place cell were modeled on the dendrite located 315.95 μm or spatially distributed 300–400 μm from the soma of the CA1 pyramidal cell, to reflect the neurons in MEC layer III synapses to the distal dendrites of the CA1 pyramidal neuron (Figs 7B and 8A) [14, 22, 37]. The spike timings of randomly selected grid cells were transformed into the excitatory postsynaptic potential (EPSP) of the place cell using the single exponential function of excitatory postsynaptic conductance (EPSG):

$$EPSG(t)=w\left(\exp\left(-\frac{t}{\tau_{decay}}\right)\right)$$ (7)

where *τ*_*decay*_ is the decay time constant (30 ms) and *w* is the synaptic conductance (200 pS) which were used in the computational CA1 PC model based on an unitary EPSP measured at the distal dendrites of CA1 PC *in vitro* [48, 49] to mimic the perforant path-evoked EPSP amplitude (~7 mV) recorded in CA1 pyramidal cells *in vitro* [50].

### Data analysis

#### Analyzing the firing rate map and spatial correlation plot

To analyze the firing rate map, the 1 m × 1 m square environment was divided into 3 cm by 3 cm bins, and the total number of spikes in each bin was divided by the total time spent in the bin [3]. The rate map was spatially smoothed with a moving boxcar window of 5 × 5 adjacent bins of each bin along both *x* and *y* axes [10]. The bin with the maximum firing rate and adjacent bins with at least 20% of the maximum firing rate were considered as firing fields [1]. We determined a place field to be viable when the size of firing field was larger than 15 adjacent bins and was smaller than 60% of the total environment size [51, 52]. To test the reliability of place field generation, we repeatedly and randomly generated grid cell inputs 100 times and calculated the proportion of viable place fields (Figs 7F, 10G–10I).

To analyze the grid characteristics, we used an autocorrelogram of the firing rate map (Figs 3C, 3F, 3I, 5 and 6) [1]. The autocorrelogram was calculated by taking the spatial correlation of the fields X and Y, where X and Y are identical to each other with the field size of M by N [3], as shown in Eq 8.

$$c(k,l)=\sum_{m=0}^{M-1}\sum_{n=0}^{N-1}X(m,n)Y(m-k,n-l),\;\left\{ \begin{array}{c} -(M-1)\leq k\leq M-1\\ -(N-1)\leq k\leq N-1 \end{array}\right.$$ (8)

For each autocorrelogram, the spacing of the grid firing field was measured from the center of the spatial autocorrelation plot to the nearest peaks around the center and averaged (Fig 4C) [1]. If there were not enough peaks near the center, the circular shape was fitted using the outermost peak [1]. To analyze the grid score, which quantifies periodicity and regularity of the grid pattern [3, 53], the autocorrelation plot was repeatedly rotated by 6° and the spatial correlation between the rotated autocorrelation plot and the original autocorrelation map was calculated as a function of rotated degree. The grid score was calculated as the difference between the minimum correlation value at 60° and 120° and the maximum correlation value at 30°, 90°, and 150° (Fig 4D).

#### The peak position of the excitatory ramp input

**Fig 11:**
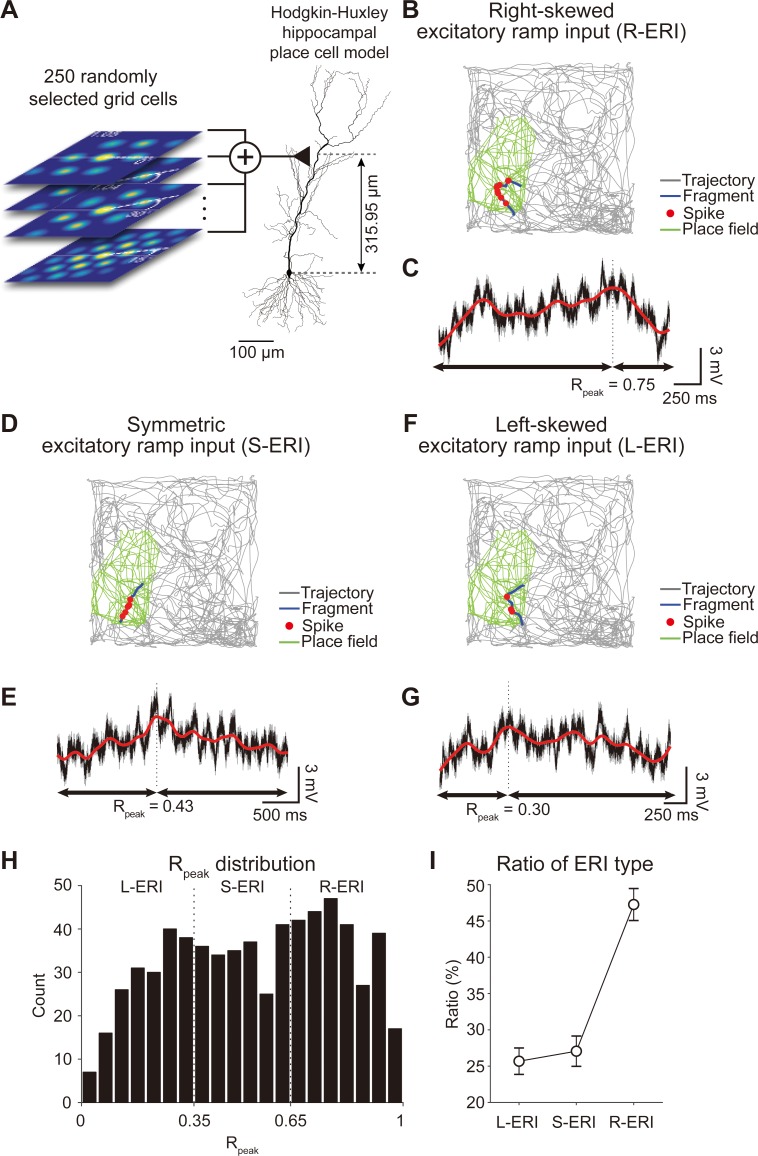
Spikes of grid cells are transformed into excitatory ramp input in place cell model. (A) 250 grid cells, each with different spatiotemporal grid field patterns, were selected from the pool of 10,000 grid cells and were used as excitatory inputs to the Hodgkin-Huxley hippocampal place cell model. (B) Spikes of place cell model (red dots) while the animal traversed (trajectory: black line) a fragment (blue line) within a place field (green). (C) Membrane voltage (*V*_*m*_, black line) of the Hodgkin-Huxley place cell model when the animal traversed the blue trajectory in (B). The spikes from 250 grid cell models are summated and transformed into a right-skewed excitatory ramp input (R-ERI) in the place cell model. The spline-fitted curve (red line) and the relative peak position (*R*_*Peak*_ = 0.75) of the R-ERI is shown. (D-E) Same figures as (B-C), but summation of excitatory input showing symmetric excitatory ramp input (S-ERI, *R*_*Peak*_ = 0.43). (F-G) Same figures as (B-C), but summation of excitatory input showing left-skewed excitatory ramp input (L-ERI, *R*_*Peak*_ = 0.30). (H) Distribution of *R*_*Peak*_ of 653 ERIs generated by 100 different sets of grid field patterns, each containing 250 spatiotemporal random grid fields patterns. ERIs are divided into L-ERI, S-ERI, and R-ERI depending on the location of *R*_*Peak*_. (I) Ratio of the number of L-ERI (25.68 ± 1.82%), S-ERI (27.06 ± 2.09%) and R-ERI (47.26 ± 2.20%) in (H).

To analyze the characteristics of place cell, we also analyzed the firing rate map of the CA1 pyramidal cell with same procedures used for the grid cell. In the smoothed firing rate map of the 1 m × 1 m square environment, we defined a “place field” as a cluster of ≥15 adjacent bins with a firing rate > 20% of the peak firing rate [54, 55]. The membrane potential of the place cell when the rat was within the place field was analyzed to characterize the shape of the excitatory ramp input (ERI), as shown in Fig 11. To classify the shapes of ERIs (Fig 11), ERI was smoothed by the Smoothing Spline method with a smoothing factor of 0.9 [56]. When the peak position of the smoothed ERI (R_peak_) was smaller than 35% of the total length of the ERI, ERI was classified as left-skewed ERI (L-ERI). When the R_peak_ was larger than 65% of the total length of ERI, ERI was classified as right-skewed ERI (R-ERI). Otherwise, ERI was classified as symmetric ERI (S-ERI).

#### Phase calculation

**Fig 12:**
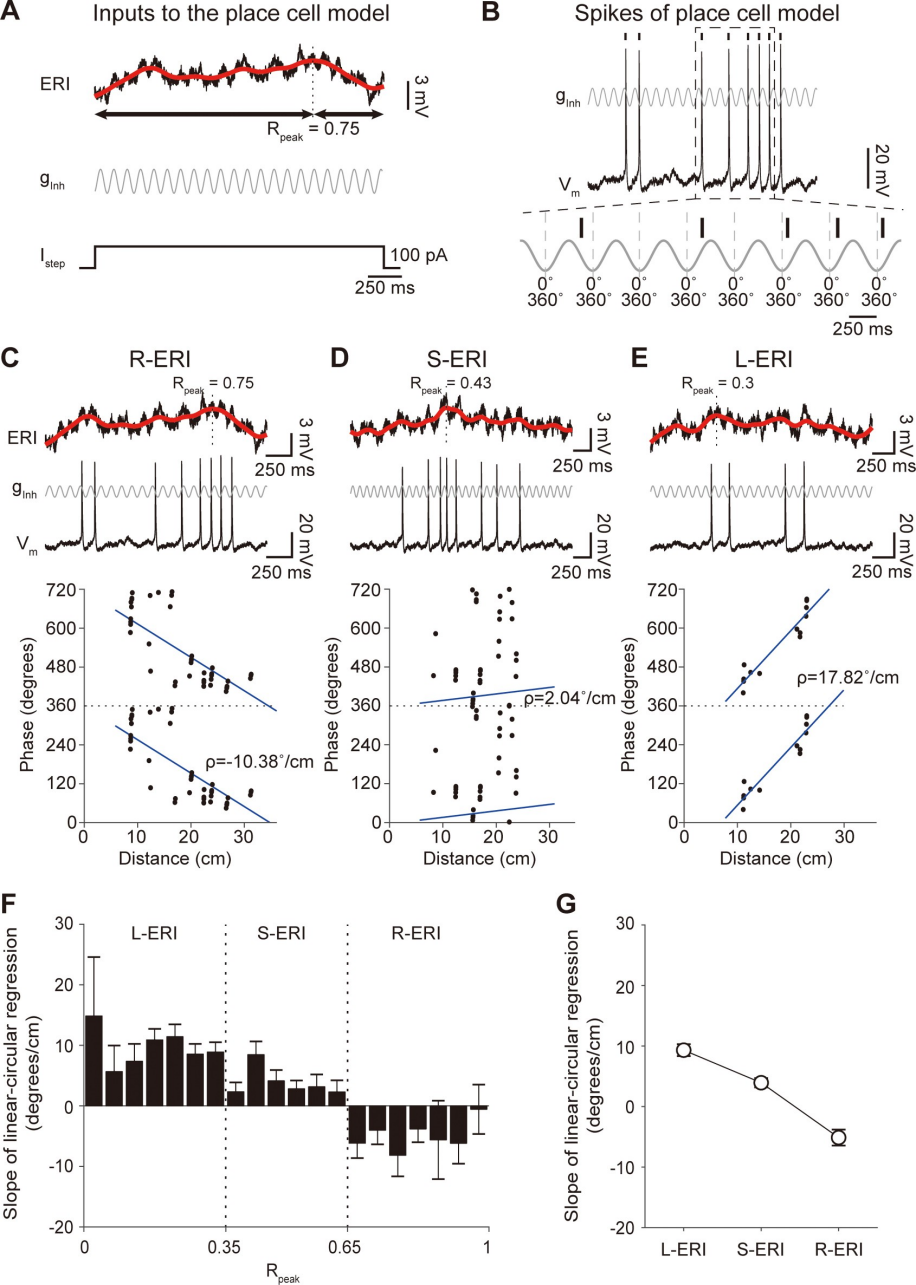
Spatiotemporally random grid cell inputs are transformed into place cell with spike phase precssion. (A) Top: Excitatory ramp input (ERI) of Hodgkin-Huxley hippocampal place cell model (black line) with spline-fitted curve (red line), which was used to determine the peak position of ERI (*R*_*Peak*_). Middle and bottom: inhibitory theta-frequency oscillation (g_*inh*_) and step current input (I_*step*_) given to place cell model. (B) Top: Membrane voltage trace of the Hodgkin-Huxley place cell model (*V*_*m*_) with g_*inh*_. Bottom: The expanded view of the dotted box above showing spike phase precession relative to g_*inh*_ from 360° to 0°. Vertical tick represents spike times and dotted lines are 0°/360° of g_*inh*_. (C-E) Top: ERI (black) with spline-fitted curve (red curve). Middle: Membrane voltage trace of the V_m_ (black) and g_*inh*_ (gray)_._ Bottom: Phase of place cell spikes (black dot) relative to g_*inh*_ plotted as a function of distance during single-pass of place field with linear-circular regression (blue line) when ERI was R-ERI (C, *ρ* = -10.38°/cm), S-ERI (D, *ρ* = 2.04°/cm), and L-ERI (E, *ρ* = 17.82°/cm). (F) Distribution of slope of linear-circular regression (*ρ*) in (C), (D) and (E) generated by 100 different sets of grid field patterns, each set containing 250 spatiotemporal random grid fields patterns. (G) Slope of linear-circular regression (*ρ*) of L-ERI (*ρ* = 9.33 ± 0.99°/cm), S-ERI (*ρ* = 3.94 ± 0.77°/cm), and R-ERI (*ρ* = -5.11 ± 1.31°/cm).

The spike phase (*Φ*) of place cell at time *t* was calculated relative to the theta- frequency inhibitory oscillation (*g*_*inh*_, Eq 6), where the peak of theta-frequency oscillation was defined as 0° or 360° (Fig 12A and 12B). *t*_1_ represents the time of the first previous peak of the background oscillation compared to the spike time, *t*, and *t*_2_ represents the time of the first background oscillation peak after the spike time, *t*.

$$\phi =360{}^{\circ}\times \frac{t-t_{1}}{t_{2}-t_{1}}$$ (9)

In analyzing the spike phase precession, the phase of the spikes elicited within the place field was plotted as distance travelled across the place field (Fig 12C–12E). To quantify the correlation between the position and the phase which arises by the phase precession phenomenon [5, 55], we calculated the slope (*ρ*) using linear-circular regression (Fig 12C–12G).

#### Statistical tests

To compare the grid characteristics of the *in vivo*-like grid cell model, the *in vivo* grid data, and the OI model, one-way ANOVA followed by Dunnett’s *post hoc* test was performed (Fig 4). A *p*-value < 0.05 was considered as statistically significant (n.s. *p*> 0.05; * *p*<0.05; ** *p*< 0.01; *** *p*<0.001).

### Simulation environment

Grid cell simulation and data analysis were conducted with MATLAB R2015a. Place cell simulation was performed with NEURON v. 7.3 [57]. The models are available on GitHub (https://github.com/kuncl/grid2place).

## Results

Grid cells in layer III of the MEC and place cells in the CA1 area of the hippocampus are anatomically only a single synapse apart, where MEC grid cells send direct excitatory synaptic inputs to the hippocampal place cells, as shown in Fig 1A and 1B. However, how the multi-peaked spike activities of grid cells that form hexagonal spike patterns in the entire environment (Fig 1C, top) are transformed into hippocampal place cell activities at specific locations (Fig 1C, bottom) is perplexing.

To investigate grid-to-place cell transformation, we constructed a neural network model composed of an anatomically and physiologically realistic place cell with grid fields generated by an OI-based grid cell model that were connected by excitatory synapses. We first developed a grid cell model that had similar spiking characteristics to the grid cells observed in *in vivo* experiments for rats navigating around a 1 m × 1 m square environment [1]. The conventional OI grid cell model simulated with grid orientation parameter *ϕ*_*VCO*_ = 30° and grid spacing/size parameter *β* = 2 generated multi-peaked grid-like spiking activities (red dots) along the rat’s trajectory (black line), as shown in Fig 2A. The firing rate map in Fig 2B, which shows the spike firing rate as a color plot, visualizes the grid fields better. Although the OI grid cell model mimicked the location and spacing between the grid fields, it exhibited an unphysiologically high peak firing rate of 323.53 Hz (Fig 2B). In fact, grid cells *in vivo* have a firing rate of around 10–20 Hz, with a maximum firing rate of up to ~ 40 Hz [1, 3]. Moreover, the firing rate of grid cell peaks at the center of the grid field as the rat moves from the edge to the center of the grid field in a Gaussian-like fashion [1, 3, 16], as seen in Fig 2C. Therefore, we incorporated these two *in vivo* features of the grid fields in the model and built an *in vivo*-like OI grid cell model that not only captures the grid field location and spacing but also the Gaussian-like grid field firing pattern for the given trajectory of the rat (Fig 2D). Fig 2E shows that our *in vivo*-like OI grid cell model exhibits a peak firing rate of 21.51 Hz.

In order to confirm that our *in vivo-*like OI grid cell model mimicked the grid cell characteristics observed *in vivo* better than the conventional OI grid cell model, our *in vivo*-like OI model was simulated with the same parameters as the conventional OI model (*β* = 2, *ϕ*_*VCO*_ = 30) and the grid field characteristics were directly compared. The raw spiking data in Fig 3A, 3D and 3G, the firing rate map in Figs 3B, 3E and 3H, and the autocorrelograms of the firing rate map in Fig 3C, 3F and 3I of our *in vivo*-like OI model, *in vivo* data, and conventional OI model were plotted, respectively. Autocorrelograms (Fig 3C, 3F and 3I) were used to analyze the grid field size, the spacing between grid fields, and the grid scores for comparison, as shown in Fig 4. We found that our *in vivo*-like OI grid cell model closely mimicked the maximum firing rate (*in vivo*-like OI model: 29.64 ± 5.50 Hz, *in vivo*: 26.53 ± 3.74 Hz, OI model: 353.37 ± 4.38 Hz; *F*_[2,6]_ = 1,668.77, *p* < 0.001; *in vivo* versus *in vivo*-like OI model, *p* > 0.05; *in vivo* versus OI model, *p* < 0.001 by Dunnett’s *post hoc* test; Fig 4A), grid field area (*in vivo*-like OI model: 373.80 ± 22.77 cm^2^, *in vivo*: 341.17 ± 24.58 cm^2^, OI model: 605.36 ± 135.65 cm^2^; *F*_[2,6]_ = 26.01, *p* < 0.01; *in vivo* versus *in vivo*-like OI model, *p* > 0.05; *in vivo* versus OI model, *p* < 0.01 by Dunnett’s *post hoc* test; Fig 4B) and grid spacing (*in vivo*-like OI model: 57.91 ± 0.07 cm, *in vivo*: 50.53 ± 5.62 cm, OI model: 68.49 ± 1.65 cm; *F*_[2,6]_ = 7.11, *p* < 0.05; *in vivo* versus *in vivo*-like OI model, *p* > 0.05; *in vivo* versus OI model, *p* < 0.05 by Dunnett’s *post hoc* test; Fig 4C) of the *in vivo*-recorded data in the OI model. Interestingly, our model had a significantly higher grid score than the *in vivo* data (*in vivo*-like model: 0.81 ± 0.09, *in vivo*: 0.43 ± 0.1, OI: 0.73 ± 0.07; *F*_[2,6]_ = 8.94, *p* < 0.05; *in vivo* versus *in vivo*-like OI model, *p* < 0.05; *in vivo* versus OI model, *p* < 0.05 by Dunnett’s *post hoc* test; Fig 4D), which quantifies the periodicity and regularity of the grid pattern [3, 53]. These results indicate that our *in vivo*-like OI grid cell model can closely capture the *in vivo* grid cell characteristics better than the conventional OI model.

The grid cell’s spike patterns *in vivo* are diverse in grid spacing, orientation, and grid field size, depending on the anatomical and electrophysiological properties of the grid cell [1, 8, 10]. Similar to *in vivo* grid cells, our *in vivo*-like grid cell model could also generate diverse grid field orientations, modulated by *ϕ*_*VCO*_ (Fig 5). Also, with *ϕ*_*VCO*_ fixed at 30°, the size of the grid field could be modulated by *β* (Fig 6A–6F). The grid field size had a negative correlation with *β* (r^2^ = 0.96, Fig 6G) and the grid field spacing was also negatively correlated with *β* (r^2^ = 0.96, Fig 6H). Based on these simulation results, we can confirm that our *in vivo*-like grid cell model could closely mimic the diversity of the grid cell firing pattern with different combinations of *β* and *ϕ*_*VCO*_.

Being only one synapse apart, how are these multi-peaked spike activities of grid cells with diverse grid patterns transformed into spike activities of hippocampal place cell at specific locations? Interestingly, anatomical studies indicate that each place cell receives approximately 100–1,000 synaptic inputs from neurons in layer III of the MEC [16, 22, 36]. Thus, we conjectured that the summation of inputs from a random collection of grid cells with diverse grid patterns may be critical in the transformation of multi-peaked grid cells to a place cell. To test our hypothesis, we first generated a pool of 10,000 grid cells, each with different grid field orientations, spacing, sizes, and peak firing rates by varying *β* and *ϕ*_*VCO*_ uniformly over the ranges 1 ≤ *β* ≤ 3.5 and 0° ≤ *ϕ*_*VCO*_ < 60°, respectively, in the *in vivo*-like grid cell model (Fig 7A). From the pool of spatiotemporally diverse grid cells, 250 grid cells were randomly selected, and their spikes were used as inputs to the full-morphology Hodgkin-Huxley hippocampal place cell model through the excitatory synapse (Fig 7B, See Materials and Methods). The number of grid cells in each parameter space (*β*, *ϕ*_*pref*_) was plotted in Fig 7C. To confirm the randomness of the selected grid cell parameter space, the ratio of entropy of parameter space was calculated as the relative entropy to the maximal entropy of parameter pool. The randomness was 100%, indicating that 250 randomly selected grid cells from the pool were perfectly spatiotemporally random and diverse (Fig 7C). Surprisingly, these 250 spatiotemporally random grid cells’ inputs to a place cell could successfully generate a place field in the place cell model of the virtual rat navigating around the environment (Fig 7D) and in the firing rate map (Fig 7E). Moreover, we repeated the simulation 100 times with 250 randomly generated grid cells and found that 94 of 100 grid input sets generated viable place fields, indicating 250 grid cells is sufficient to reliably generate a place field (Fig 7F).

Biologically, the real synapses from MEC grid cells to hippocampal CA1 place cells are spatially distributed at dendritic locations of 300–400 μm from the soma of the CA1 pyramidal cell in the hippocampus [14, 22, 37]. When we tested whether 250 grid cell inputs onto distal dendritic synapses that were spatially distributed could generate grid cell-to-place cell transformation (Fig 8A), we found that 250 spatiotemporally random grid cells to a place cell could successfully generate a place field (Fig 8B) and firing rate map (Fig 8C).

One important result of our simulation is that, when we repeated the simulation with two different sets of 250 spatiotemporally random grid cells with different (*β*, *ϕ*_*pref*_) parameters, as in Fig 9A and in Fig 9D, the transformed place field was generated at a completely different location (Fig 9B, 9C, 9E and 9F) to that in Fig 7D and 7E. Such simulation results are related to the *in vivo* observation, where optogenetic modulation or perturbation of grid cell activities changes the place field location within the same environment [58–60].

When we repeated the simulation with a set of 100 spatiotemporally random grid cells selected from the pool (Fig 10A), we found that an insufficient number of spikes were evoked in the place cell (Fig 10B and 10C), whereas inputs from 500 spatiotemporally random grid cells selected from the pool (Fig 10D) resulted in too much excitation in the place cell (Fig 10E and 10F), both failing to transform grid cells to place cell. When we systematically increased the number of grid cells from 50 to 500 with an increment of 50 grid cells, we found that the number of spatiotemporally random grid cells for transformation to viable place cells in firing rate and place field size was optimal with grid cell numbers in the range of 250 to 300 (Fig 10G–10I). These results suggest that a certain range of grid cell numbers is required for successful grid-to-place cell transformation and that the number of grid cells required is well within the lower range of the anatomical connections observed in the MEC-CA1, which is around 100–1,000 grid cells [16, 22, 36].

The other spatial feature of place cells, in addition to having place fields at specific locations, is that they show the spike phase precession phenomenon, where the spike phases of place cells advance relative to the ongoing theta-frequency oscillation when a rat traverses a place field [6, 55]. Moreover, *in vivo* whole-cell patch clamp studies demonstrated that a place cell’s membrane potential shows a depolarizing excitatory ramp-like input (ERI) shape as the rat passes through the place field [31]. Indeed, *in vitro* and computational modeling studies confirm that ERI is required for spike phase precession to occur [33, 61]. Therefore, for our grid-to-place cell transformation to be realistic, our model should be able to replicate the spike phase precession phenomenon with ERI as well. In our neural network model, we observed that the 250 spatiotemporally random grid cell patterns used as inputs to the CA1 pyramidal neuron model (Fig 11A) robustly generated a place field (Fig 11B, green area). As the rat traverses through a specific fragment of the trajectory within the place field (Fig 11B, blue line), the place cell receives ERIs as inputs from grid cells (Fig 11C). We found that, depending on the part of the place field the rat is traversing (Fig 11B, 11D and 11F), different shapes of ERIs arose: right-skewed ERI (R-ERI, Fig 11C), symmetric ERI (S-ERI, Fig 11E), and left-skewed ERI (L-ERI, Fig 11G). By generating 100 different sets of grid field patterns, each set containing 250 spatiotemporal random grid fields patterns, we obtained 653 ERIs from 94 viable place fields. Among these ERIs, analyzing the distribution of ERI peaks revealed that R-ERI most frequently occurs compared to S-ERI and L-ERI (Fig 11H and 11I), which is similar to what is observed *in vivo* [31].

Finally, to confirm that the ERIs are directly related to spike phase precession in place cells, ERI was superimposed with theta-frequency (10 Hz) background inhibitory oscillatory conductance (*g*_*inh*_), step current, and Gaussian white noise in the place cell model (Fig 12A and 12B), to replicate the *in vivo*-observed background oscillation in place cells [6, 13, 42, 43]. When spike phases relative to the given *g*_*inh*_ were analyzed, robust spike phase advancement was observed when R-ERI was superimposed with *g*_*inh*_ (Fig 12C, negative linear-circular regression (*ρ*) while S-ERI and L-ERI could not generate spike phase advancement (Fig 12D and 12E), which was also confirmed by analyzing the distribution of slope of linear-circular regression (*ρ*) between spike phase and distance (Fig 12F and 12G). Thus, we demonstrate that the spatiotemporally random and diverse grid spike patterns generate grid cell spikes that transformed into R-ERI in the place cell model that could replicate the spike phase precession phenomenon.

## Discussion

In this study, we built a physiologically and anatomically realistic neural network model consisting of a Hodgkin-Huxley place cell model with excitatory synapses receiving *in vivo*-like grid fields from *in vivo*-like OI grid cell model. Using this model, we demonstrated that the integration of inputs from grid cells that have spatiotemporally random and diverse multi-peaked grid spike patterns as inputs to place cells can successfully perform grid-to-place cell transformation (Fig 7). In addition, such input requirements in our neural network model allowed us, for the first time, to simultaneously capture one more important *in vivo* characteristics of place cells: the spike phase precession phenomenon [6, 55] (Fig 12). The results from our model indicate that integration of random and diverse grid cell input patterns as input to place cells is critical for the spatial information transformation in the entorhinal-hippocampal network *in vivo*.

In our simulation, the optimal number of grid cells (250–300 grid cells), each with completely random and diverse distribution of grid spacing, orientation, and phase, can aid grid-to-place cell transformation (Figs 7–10). The numbers of afferent inputs derived from our simulation are in agreement with the anatomically and physiologically realistic estimate of the number of synaptic connections between MEC grid cells and place cells [14, 22] where MEC grid cells with different grid field sizes, spacing, orientations, and phases [1, 3, 10] in layer III of the MEC make up to 1,000 direct synaptic connections to excitatory neurons in the CA1 area of the hippocampus [16, 22, 36]. However, caution is warranted in interpreting these results since the number of grid cells specified as 250–300 is only valid for our simulation parameter and cannot be generalized *in vivo*. The synapse model we used in our study was fixed to have maximal synaptic conductance of 200 pS, while synaptic conductance *in vivo* may vary depending on the distance between the soma and the input locations [48], laminar location, recruitment of inhibitory synapses [62], number of inputs, and neuromodulatory state [63]. Also, since our model gave all inputs from grid cells into one single synapse on the dendrite of the place cell (Fig 7B) or spatially distributed synapses (Fig 8A), the number of grid cell inputs we suggest in our model should only be used as a guide in gauging the relative contribution of low and high synaptic conductance in grid-to-place cell transformation.

The most important novelty of our study is that we developed a network model of a place cell with grid cell inputs that could, for the first time, capture not only the grid-to-place cell transformation but also the spike phase precession phenomenon observed in place cells (Fig 12). Our simulation result, that grid cell inputs can generate spike phase precession in place cells, is in line with experimental observations [17, 64, 65], suggesting that synaptic input from the MEC may be critical for the phase precession. Many previous computational studies solely focus on demonstrating the transformation of periodic hexagonal spike activities of MEC grid cells to non-periodic single place field in hippocampal place cells [15, 16, 23–30]; especially grid cells with uniform distribution of orientation, phase, and spacing [30], and grid cells with random variability only in spacing and orientations but not phase [16] or grid cells with random connectivity by a competitive Hebbian learning process with variability in orientation, phase, and spacing [27]. However, none of the transformed place cells showed spike phase precession. It was evident from other *in vivo*, *in vitro*, and *in silico* studies that emergence of R-ERI is critical in generating spike phase precession within the place field [31, 33, 61] and afferent grid cell inputs to place cells should integrate in the form of EPSP to form R-ERI. In our simulation, integrated synaptic EPSPs evoked by grid cell inputs successfully generated both R-ERI and the spike phase precession phenomenon (Figs 11 and 12). It is well established that such synaptic integration is affected by the passive dendritic cable properties of the neuronal membrane [66] as well as by active dendritic properties, such as voltage-gated Ca^2+^, Na^+^ ion channels [67], *I*_*h*_ [68] and *I*_*M*_ [41]. Therefore, we included these *I*_*Na*_, *I*_*h*_, and *I*_*M*_ ion channels into our multi-compartment full-morphology Hodgkin-Huxley CA1 pyramidal neuron and the synapse between the grid cell and the place cell was modeled to reflect the anatomical detail that afferent grid cell inputs arrive in the distal dendrites of the CA1 pyramidal neuron. Through such physiologically and anatomically realistic modeling of the synapse between grid cell and place cell, we could successfully demonstrate grid-to-place cell transformation with spike phase precession phenomenon. However, many previous studies modeled place cells as integrate-and-fire neurons [30], simplified spiking units [25, 26], or even non-spiking units [16, 35], where the place cell model simply summed the firing rates of grid cells, which may be the reason they failed to replicate the spike phase precession phenomenon. Moreover, it is well established that anatomical location of grid cells along the dorsoventral axis of the MEC correlates to the various spatial scale of grid field, and dorsoventral place cells are correlated with place field size [8, 69]. Although our network model included a physiologically and anatomically realistic synapse model between grid cells and a place cell, our model is limited by the fact that place cells receive inputs from grid cells showing all variations of grid cell activity along the dorsoventral axis [8]. Moreover, recent experimental evidence suggests that cooperative inputs from both the MEC and CA3 regions are important to control phase precession [64, 65]. Therefore, further investigation on the role of distinct connectivity between dorsal/ventral MEC-to-CA1 region and CA3-to-CA1 region on the grid cell-to-place cell transformation will be necded.

Our simulation results also could capture a recent *in vivo* observation, where the place field of the same place cell changes location within the same environment when grid cells in the MEC were optogenetically perturbed through partial inactivation or depolarization [58, 60] without changing their grid field firing locations [59]. Whenever we repeated the random selection of 250 grid cells from the pool, the place field was generated at a different location (Fig 9B and 9E) and the number of grid cells with specific angle and spacing combinations were completely random (Fig 9A and 9D).

Overall, our results show that an anatomically and physiologically realistic network model of grid cells and place cell can, for the first time, closely simulate key features of *in vivo*-observed grid-to-place cell transformation. Also, our model provides evidence that the dynamic integration of spatiotemporally random and diverse spiking activities in spatially-selective neurons may hold the key to unraveling the mechanisms underlying spatial navigation in our brains.
